# Clinical Risk Factors for Gastroduodenal Ulcer in Romanian Low-Dose Aspirin Consumers

**DOI:** 10.1155/2016/7230626

**Published:** 2016-08-08

**Authors:** Anca Negovan, Mihaela Iancu, Valeriu Moldovan, Septimiu Voidazan, Simona Bataga, Monica Pantea, Kinga Sarkany, Cristina Tatar, Simona Mocan, Claudia Banescu

**Affiliations:** ^1^University of Medicine and Pharmacy, Tirgu Mureș, Gheorghe Marinescu 38, 540139 Mures, Romania; ^2^University of Medicine and Pharmacy “Iuliu Hațieganu” Cluj-Napoca, 8 Victor Babeş, 400012 Cluj-Napoca, Romania; ^3^Emergency County Hospital, Tirgu Mures, Gheorghe Marinescu 50, 540136 Mures, Romania

## Abstract

*Background*. Aspirin use for cardiovascular or cancer prevention is limited due to its gastrointestinal side effects.* Objective*. Our prospective, observational case-control study aims to identify the predictive factors for ulcers in low-dose aspirin consumers (75–325 mg/day).* Methods*. The study included patients who underwent an upper digestive endoscopy and took low-dose aspirin treatment.* Results*. We recruited 51 patients with ulcer (ulcer group) and 108 patients with no mucosal lesions (control group). In univariate analysis, factors significantly associated with ulcers were male gender (*p* = 0.001), anticoagulants (*p* = 0.029), nonsteroidal anti-inflammatory drugs (*p* = 0.013), heart failure (*p* = 0.007), liver (*p* = 0.011) or cerebrovascular disease (*p* = 0.004), diabetes mellitus (*p* = 0.043), ulcer history (*p* = 0.044), and alcohol consumption (*p* = 0.018), but not* Helicobacter pylori* infection (*p* = 0.2). According to our multivariate regression analysis results, history of peptic ulcer (OR 3.07, 95% CI 1.06–8.86), cotreatment with NSAIDs (OR 8, 95% CI 2.09–30.58) or anticoagulants (OR 4.85, 95% CI 1.33–17.68), male gender (OR 5.2, 95% CI 1.77–15.34), and stroke (OR 7.27, 95% CI 1.40–37.74) remained predictors for ulcer on endoscopy.* Conclusions*. Concomitant use of NSAIDs or anticoagulants, comorbidities (cerebrovascular disease), and male gender are the most important independent risk factors for ulcer on endoscopy in low-dose aspirin consumers, in a population with a high prevalence of* H. pylori* infection.

## 1. Introduction

The use of low-dose aspirin (LDA, 75–325 mg/day) has continually increased during recent decades [1]. Beside its cardiovascular effect, aspirin has been proved to be beneficial for cancer prevention, which probably further increases its use [2]. The risk of harmful side effects, especially gastrointestinal (GI), limits the general benefit of aspirin use [3]. LDA decreases the incidence of cardiovascular events by 12% [1] but the incidence of serious GI adverse events is approximately one case per 1000 persons/year in overall population [2]. Despite its relatively low risk for GI bleeding, the millions of aspirin users worldwide determine an important increase in the number of drug related GI complications [4].

In order to minimize the risk of bleeding among patients treated with LDA a number of recommendations were developed by interdisciplinary consensus groups [5]. Thus, the antiplatelet therapy risk factors for GI events, namely, history of ulcer disease,* Helicobacter pylori* (*H. pylori*) infection, age > 70 years, and concomitant use of nonsteroidal anti-inflammatory drugs (NSAIDs) or other antithrombotic drugs were defined and protective strategies were recommended [5, 6]. Data regarding other possible factors which can increase the risk of GI bleeding in LDA-treated patients are controversial: comorbidities, male gender, use of calcium channel blockers, smoking, alcohol abuse, obesity, aspirin regimen, or formulation [7, 8].

Clinical and epidemiological data from Eastern Europe regarding GI complications in patients treated with LDA are scarce and inconsistent. The previously mentioned risk factors may also be positively influenced by the general characteristics of the population, the increased prevalence of* H. pylori* infection, and genetics and by the differences in prevalence of various gastritis phenotypes [3].

## 2. Scope

Our study aims to determine the most important predictive factors for gastroduodenal ulcer in LDA-treated patients.

## 3. Methods

The study included consecutive patients admitted to the 3rd Medical Clinic in Tirgu Mures, Romania, who underwent an upper digestive endoscopy between January 2010 and December 2014 and who were under chronic LDA-treatment without concomitant protective therapy (PPI). The ethical committee of the University of Medicine and Pharmacy of Tirgu Mures, Romania, approved this study.

Patients who were included attended endoscopy for digestive symptoms or anemia or for screening before a cardiovascular surgery. A written consent was obtained from every patient. We considered LDA exposure as daily administration of 75 mg, 100 mg, or 125 mg of aspirin (available formulated aspirin doses in Romania) for at least one month prior to investigation.

Demographic and clinical data were collected from each patient. We registered the symptoms as the reason for endoscopy recommendation (upper abdominal pain, heartburn, nausea, vomiting, and bloating). We investigated the history of dyspeptic symptoms and the diagnosis of prior peptic ulcer (clinical, radiological, or endoscopic diagnosis) in every patient. To investigate drug exposure, we used a structured interview and medical records. We recorded concomitant use of other potential gastrotoxic drugs: NSAIDs, acenocumarolum, and low-weight molecular heparin (LWMH) as daily administration of a regular dose for at least two weeks prior to endoscopy. We used the available medical records to check for medical prescriptions and comorbidities (hypertension, ischemic heart disease, valvular disease, arrhythmias, heart failure, cerebrovascular disease, respiratory disease, renal disease, liver disease, and diabetes). We excluded patients with severe medical conditions/end-stage disease (severe cardiac failure, malignant disease, severe renal insufficiency, severe respiratory diseases, Child-Pugh C stages of cirrhosis, and severe dementia) evaluated on endoscopy especially for suspicion of upper digestive occult bleeding. They were excluded if the clinical status did not allow us to conclude the investigation, to obtain all biopsies during endoscopy, or to finish the interview. Other exclusion criteria included patients taking clopidogrelum or newer oral anticoagulants (dabigatran, apixaban, and rivaroxaban) as well as patients treated with systemic corticosteroid therapy. The low number of patients taking concomitant clopidogrelum (3 patients), new antithrombotic therapy (3 patients on non-anti-vitamin K therapy), or systemic corticotherapy (4 patients on methylprednisolone therapy) did not allow us to study these drugs as independent risk factors for ulcer in aspirin consumers.

A single endoscopist blinded to drug exposure and symptoms carefully examined the gastric and duodenal mucosa. Mucosal defects larger than 5 mm and extended into the deeper layers of the gastric or duodenal wall were defined as ulcer. Patients with gastroduodenal surgery, varices, and active severe bleeding or patients in whom a gastric cancer was discovered on endoscopy were excluded.

During the upper digestive endoscopy two biopsy specimens from the antrum and two from the corpus (from lesser and greater curvatures) were taken for routine histology and were examined by a single pathologist blinded to drug exposure and symptoms.* H. pylori* infection was considered negative if* H. pylori* was absent from all biopsy sites and positive if at least one histology test was positive.

All the collected data were recorded in a specially designed database.

### 3.1. Statistical Analysis

Qualitative nominal variables were presented using absolute frequencies (number of cases) and relative frequencies (%). Chi-square or Fisher's exact tests were performed for the analysis of associations between possible predictors for gastroduodenal ulcer occurrence in patients taking long term LDA:* H. pylori *infection, concomitant use of other antithrombotic drugs or NSAIDs, history of complicated or uncomplicated peptic ulcer disease, comorbidities, male gender, age, symptoms, alcohol consumption, and smoking. We compared the frequencies of all these factors in ulcer group (*n* = 51) and no lesions-group (patients without endoscopic lesions, *n* = 108). We did not take into account for this study data from patients with erosions or petechiae (*n* = 82). Using the guidelines defined in G^*∗*^Power 3.1.9.2 [9], a desired sample size of 131 subjects was obtained for an alpha level of 0.05, a power of 0.80, and a large effect size OR = 3. Based on these arguments, we considered taking into account the patients with negative endoscopy (without patients with mild endoscopic lesions) as nonevent cases, assuring us a balanced design and it was sufficient to develop an accurate prognostic model. The odds ratio (OR) with 95% confidence interval was also calculated to assess the intensity of associations with gastroduodenal ulcer in patients taking LDA. Univariate binomial logistic regression was used to test and estimate the individual effect of the studied predictor factors (such as hematemesis), with a frequency below 5. All significant factors and factors whose unadjusted estimated significance level was *p* < 0.25 in univariate regression were potential candidates for multivariate logistic regression, used to describe the independent risk factors for gastroduodenal ulcer. The final model development was based on multiple nested model comparisons using Chi-square difference testing (Likelihood ratio test). Performance of the final model was evaluated with C-statistic by measuring the area under the receiver-operating characteristic curve. Limits of the 95% confidence interval for C-statistic were also generated to give estimates of precision. Goodness of model fit was tested for statistical significance using the Hosmer and Lemeshow test.

Statistical significance for all bilateral tests was accomplished when the estimated significance level *p* was lower than 0.05.

Statistical analysis was realized with the advanced software environment for statistical computing and graphics, R version 3.2.2 (R Foundation for Statistical Computing, Vienna, Austria).

## 4. Results

**Figure 1:**
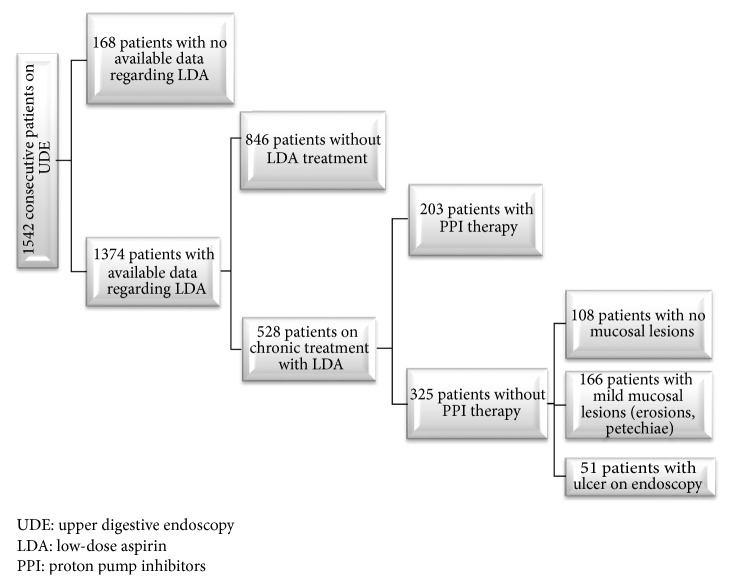
Flowchart regarding patients' selection.

From the total number of 1542 consecutive patients who attended an upper digestive endoscopy, 1374 had available data regarding aspirin consumption. A number of 528 patients were treated with LDA, from whom 325 were without PPI cotreatment. We finally recruited 159 patients: 51 patients with ulcer on endoscopy (ulcer group) and 108 patients with no gastroduodenal mucosal lesions on endoscopy (control group) (Figure 1).

### 4.1. Patient Background

A number of 63 (39.6%) patients were free of digestive symptoms before endoscopy. Combined antithrombotic therapy (aspirin plus anticoagulants) was recorded in 30 patients: 23 patients (14.4%) had oral anticoagulants (acenocumarolum, ceased 24–48 hours before endoscopy for an INR (International Normalized Ratio) <1.5 tested in the morning of investigation) and 7 patients (4.4%) had cotreatment with LMWH.

Regarding the history of ulcer, 46 patients (28.9%) described at least one episode of upper abdominal pain (ulcer-like) which required seeking care and specific treatment in their past medical history (including* H. pylori *eradication therapy), with no imagistic investigations. Only 12 patients (7,5%) had a history of peptic ulcer confirmed on radiology or endoscopy and other 5 patients (3.1%) had previous episodes of upper digestive bleeding.

### 4.2. Bivariate Analysis

Between the ulcer group and no lesion group gender showed a statistically significant difference (Table 1), whereas older age (>70 y) did not. According to our results, the history of ulcer was significantly associated with a new gastroduodenal ulcer in patients taking LDA (OR = 2.45, 95% IC: 1.16–5.19). The presence of* H. pylori *infection was found not to be significantly associated with ulcer in our aspirin consumer population, while concomitant use of anticoagulants or NSAIDs was a significant risk factor for ulcer (OR = 3.00, 95% IC: 1.32–6.79).

Concomitant diseases, heart failure, stroke, diabetes, kidney disease, or liver disease were statistically associated with a high frequency of ulcer. None of the digestive symptoms were found to be predictive for ulcer in LDA aspirin consumers. Alcohol consumption showed a significant association with ulcer in LDA consumers, while smoking (over 5 cigarettes/day) did not.

### 4.3. Multiple Binary Logistic Regression

From all considered predictors for GI ulcers in LDA consumers, it was found that gender, cotreatment with NSAID or antithrombotics, history of peptic ulcer, comorbidities (heart failure, diabetes, liver, and cerebrovascular disease), and alcohol consumption were positively associated with the presence of gastroduodenal ulcer in patients taking LDA in the univariate binary logistic analysis (Table 2).

According to our multivariate regression analysis results, the history of peptic ulcer, concomitant treatment with NSAIDs or anticoagulants, male gender, and cerebrovascular disease remained factors with a significant positive effect on endoscopic ulcer and can be considered as independent risk factors in LDA aspirin consumers for our population (Table 3). Regarding the values of estimated unstandardized regression coefficients of the final model, the most important factors positively associated with gastroduodenal ulcer were NSAIDs and cerebrovascular disease. The odds of gastroduodenal ulcer increased by 8.0 (95% CI, 2.09 to 30.58) for NSAIDs consumers and by 7.27 (95% CI, 1.40 to 37.74) in the case of patients with cerebrovascular disease, effects adjusted for other covariates. Liver disease tended to have a positive effect on ulcer in LDA consumers but did not achieve the statistical significance.

According to this logistic model, 70.69% of gastric ulcer patients were correctly classified along with 81.6% of patients without gastric lesions, resulting on average that, for a 30% risk of gastric ulcer, a proportion of 78.2% (95% CI: 69%–85%) of patients were correctly classified. Estimates of Brier coefficients, R2, indicated a good suitability of the model to the database; Somers coefficient, c, statistics equivalent to the area under the ROC-AUROC curve showed a good ability to discriminate between patients with gastric ulcer and those with no present injuries (Table 4).

## 5. Discussion

Data regarding aspirin consumption and GI events arising from the eastern part of Europe are not consistent, as well as data regarding its nonbleeding gastrointestinal side effects. Patients on PPI therapy were excluded as previous administration of protective therapy can influence the endoscopic findings. The aim of our present study was to investigate the interplay between gastrotoxic therapy (aspirin, NSAID, and anticoagulants) and* H. pylori* infection without the protective effect of PPI. We investigated the risk factors for ulcer in patients with low-dose aspirin in order to identify possible protective strategies for a population with a high prevalence of* H. pylori* infection.

The frequency of ulcers irrespective of localization (duodenal or gastric, 23%) was higher in our study than that reported in Asian population: 6.2% in Kawamura et al. study [10] or 18.8% in Nema et al.'s study [11]. The percentage of ulcer in Western patients without other gastrotoxic medications except for aspirin is also generally lower (Yeomans et al. 11%) [12].

Despite male gender not being recognized as a risk factor for bleeding ulcer in LDA consumers according to current research [7], it was associated with an increased risk for ulcer on endoscopy (OR = 5.20, 95% CI = 1.77–15.34) in our population. An increased frequency of ulcer in male patients taking LDA can be observed in both Asian and Western studies, but the difference is usually not significant [11, 12]. We could not explain the magnitude of risk for ulcer in male patients in our study, except for the high frequency of ulcer in the duodenum (21 patients with duodenal from 51 ulcer patients, data not showed) in comparison with other studies (2 patients with duodenal ulcer from 38 ulcer patients in Shiotani et al.'s study) [13, 14]. The high prevalence of* H. pylori* infection in our area and previous observations that sustained theincreased risk for duodenal ulcer in* H. pylori* positive male patients taking NSAIDs compared to females [15] may be an explanation for our findings.

Although patients with ulcers tend to be older than patients with normal mucosa, the difference was not statistically significant in our study. The vulnerability of aged mucosa on aspirin aggression [16] may be balanced by a decreased acid secretion in the gastric mucosa with gastric atrophy or/and intestinal metaplasia after longtime evolution of* H. pylori *infection [17] or by an increased mucosal tolerance to chronic drug exposure.

Interaction between aspirin and* H. pylori* in the upper digestive tract is still a matter of debate [18, 19]. Many studies have found that* H. pylori* infection in LDA consumers increases the risk for GI bleeding in patients treated with LDA (OR = 4.7; 95% CI: 2.0–10.9 in Lanas et al.'s study) [20]. There have also been systematic reviews that failed to clearly sustain this observation, maybe due to inhomogeneous studies conducted in different populations [21]. Regarding endoscopic lesions, our study did not reveal a significantly increased risk for gastroduodenal ulcer in* H. pylori* positive patients (50% with ulcer versus 36.5% without lesions, *p* = 0.11, OR = 1.73, 95% CI: 0.87–3.43). The frequency of* H. pylori* positive and negative patients with ulcer in our study (39.6% versus 11.9%) was different, but comparable with similar reported data in European patients (37% versus 16% in Pilotto et al. study) [22] but very different from that reported in Asian studies (8.4% versus 4.6% in Uemura et al.'s study) [23]. Our logistic regression models failed to prove* H. pylori* infection as an independent risk factor for ulcer in LDA consumers as the majority of Western studies did. Gastric acid secretion seems to play an important role in aspirin related lesions occurrence. The lack of association between* H. pylori* infection and aspirin related ulcer was observed more frequently in the Asian population than in Western populations [22, 24]. Decreasing acid secretion in* H. pylori* infected patients in the Asian population, and probably in the Romanian population, and genetic differences could be the reasons for our findings [25]. The interplay of systemic and local mechanisms of gastric injury and aspects related to the “age” and phenotype of the* H. pylori *infection can explain the different study results in Asian or Western populations [25], and probably our authentic results are rather “in-between” due to the frequency of* H. pylori* infection and geographical position of our country [26]. There are no recently published studies regarding the overall prevalence of* H. pylori* infection in the Romanian population. To the best of our knowledge the latest data reported a 68,5% infection prevalence in general adult population in the Western region of the country [27], one of the highest reported in Europe [26].

The majority of studies demonstrated that a history of complicated or uncomplicated ulcer leads to a twofold or threefold increase in the risk for bleeding compared to nonbleeding, in LDA users [21, 28]. One of the underlying mechanisms seems to be related to scar vulnerability and probably the persistence of* H. pylori* infection in patients with previous ulcers. In our study, prior ulcer diagnosis was an independent risk factor for a new ulcer on endoscopy, increasing the risk by three times (OR = 3.07, 95% CI: 1.06–8.86, *p* = 0.038), similar to the vast majority of studies.

Despite the poor definition of severe comorbidities and difficulties to establish criteria for their presence or severity, some studies investigated their role as risk factors for GI bleeding, more frequently for NSAIDs than for aspirin users [7]. The role of extradigestive comorbidities as independent risk factors for bleeding ulcer has been demonstrated also in a recent epidemiological study [29] and may be related to many other subtle systemic pathological mechanisms. Data are sparse and not conceivable for aspirin users, only hypertension, severe left heart failure, and renal failure increasing the risk for bleeding in a cohort study on aspirin users [30]. We did not study hypertension as a predictor for ulcer occurrence in aspirin users, as the majority of our patients (81%) presented this condition for aspirin recommendation. On multivariate analysis, history of stroke, heart failure (irrespective of functional class), and liver disease remained predictors for ulcer, but the strongest predictor for ulcer on endoscopy was cerebrovascular disease (*p* = 0.018, OR 7.27, 95% CI: 1.40–37.74). Our findings underlined the role of protective strategies in selected elderly LDA consumers, as the etiology of bleeding episodes has shifted during the past years from* H. pylori* infection towards LDA consumption and related comorbidities [31].

In certain studies that investigated bleeding in aspirin users, alcohol was found to be a risk factor if the prevalence of alcohol consumption was lower (15.1%) [32] while it had no influence if the frequency of alcohol consumers was higher in the studied groups (30.2%) [7, 33]. With a percentage of 18.8% of alcohol consumers, our study also identified alcohol use as a risk factor for ulcer on endoscopy, using multiple logistic regressions (OR = 3.0, 95% CI: 1.2–7.4). Despite the proven role of smoking in the pathogenic mechanism of peptic ulcer [34] that may be synergistic with LDA mucosal aggression and* H. pylori* inflammation, smoking was not a predictor for ulcer in our group, as in other published studies [7]. Alcohol consumption and smoking, although involved in the pathogenesis and progression of many diseases, did not have a predictive role for ulcer in our final model study after adjustment for the presence of comorbidities or concomitant gastrotoxic drug consumption.

Dyspeptic symptoms were not correlated with ulcers on endoscopy in our group, as the majority of published data would indicate [12, 35]. Our findings underlined the lack of premonitory symptoms before major complications like bleeding or perforation in patients with LDA related ulcer and the importance of specific risk factors' identification. We did not study bleeding complications of aspirin in this work, but ulcer as a good surrogate marker for bleeding [36], as was proved in other work. Benign symptoms (dyspeptic) were not correlated with the presence of ulcer that is a frequent forerunner for bleeding or perforation.

A highly increased risk for ulcer was calculated in patients with concomitant LMWH or acenocumarolum administration (OR = 4.85, 95% CI: 1.3–17.6, *p* = 0.01). There are published research that reported an increased number of bleeding related to aspirin plus anticoagulants compared to aspirin alone, not giving a relative risk and none studying acenocumarolum [7]. A high percentage of patients (18.8%) had combined antithrombotic therapy that allowed us to investigate the risk. The risk magnitude was surprisingly higher as our end-point was ulcer not bleeding, and we did not include patients with active upper digestive bleeding. The small bleeding promoted by anticoagulants and the delay in healing of* H. pylori* or aspirin induced lesions can explain these observations.

As the vast majority of studies has proved [7, 9] concomitant use of NSAIDs with LDA is a significant risk factor for gastroduodenal mucosal injury, increasing the risk for bleeding threefold compared to aspirin use alone. We calculated a higher risk for ulcer on endoscopy (OR = 8.00, 95% CI: 2.09–30.5) in patients with concomitant use of NSAIDs and LDA.

Despite its disadvantages (a study performed on an “endoscopic population”) with possible selection bias, our study revealed the predictive value of the most important risk factors for ulcer on endoscopy in a population with a high prevalence of* H. pylori* infection. Data from our study can be used to develop specific preventive strategies for an aging population with many comorbidities and cotreatments, with a high prevalence of ulcer on endoscopy.

## 6. Conclusions

Concomitant use of NSAIDs or anticoagulants, comorbidities (especially cerebrovascular disease), and male gender are the most important independent risk factors for ulcer on endoscopy in patients treated with low-dose aspirin, in a population with a high prevalence of* H. pylori* infection.

## Figures and Tables

**Table 1:** Group differences regarding studied factors in patients treated with low-dose aspirin.

Variables	Ulcer-group (*n*_1_ = 51)		No lesion-group (*n*_2_ = 108)		*p* ^a^	OR	95% CI
	No.	%	No.	%			
Male gender	35	68.6	53	49.1	**0.02**	0.44	0.21–0.88
Age > 70	20	39.2	43	39.8	0.94	0.97	0.49–1.42
Peptic ulcer history	26	61.9	37	39.8	**0.01**	2.45	1.16–5.19
*H. pylori* positive	25	50.0	38	36.5	0.11	1.73	0.87–3.43
Anticoagulants	16	31.4	14	13.2	**0.007**	3.00	1.32–6.79
NSAIDs	12	25.0	9	8.9	**0.008**	3.40	1.32–8.77
Heart failure	38	82.6	52	60.5	**0.009**	3.10	1.29–7.46
Cerebrovascular disease	13	28.3	4	4.5	**<0.001**	8.27	2.51–27.21
Diabetes mellitus	21	44.7	19	22.4	**0.008**	2.80	1.30–6.05
Kidney disease	15	32.6	13	14.8	**0.01**	2.79	1.19–6.54
Liver disease	27	62.8	34	40.5	**0.01**	2.48	1.16–5.28
Respiratory disease	12	28.6	16	18.4	0.18	1.77	0.75–4.20
Upper abdominal pain	19	41.3	43	42.6	0.88	0.94	0.46–1.92
Nausea/vomiting	9	20.5	10	10.1	0.09	2.28	0.85–6.10
Heartburn	8	18.2	23	23.0	0.51	0.74	0.30–1.82
Regurgitation	2	4.5	7	7.0	0.09	2.28	0.85–6.10
Bloating	14	31.8	18	18.2	0.07	2.10	0.93–4.74
Smoking^b^	5	13.2	7	8.1	0.51	1.71	0.50–5.77
Alcohol consumption^c^	15	39.5	15	17.4	**0.008**	3.08	1.31–7.26

^a^Obtained from Chi-square or Fisher's exact tests.

^b^Over 5 cigarettes/day.

^c^More than 2 units/day, 1 unit = 10 mL pure alcohol.

OR: odds ratio.

CI: 95% confidence interval.

NSAIDs: nonsteroidal anti-inflammatory drugs.

**Table 2:** Results from univariate binary logistic regression.

Variables	Statistics *Z*	*p* ^a^	Crude OR	95% CI
Gender M versus F	2.29	**0.001**	4.11	1.72–9.85
Age (years)	0.50	0.472	1.01	0.98–1.05
Age ≥ 70 years	−0.07	0.942	0.98	0.49–1.93
Anticoagulants	2.64	**0.029**	3.10	1.13–8.55
NSAIDs	2.54	**0.013**	3.56	1.31–9.68
Heart failure	2.53	**0.007**	4.22	1.47–12.09
Kidney disease	2.36	0.07	2.46	0.93–6.53
Respiratory disease	1.31	0.07	2.46	0.93–6.53
Liver disease	2.36	**0.011**	2.97	1.28–6.90
Cerebrovascular disease	3.48	**0.004**	6.48	1.83–22.90
Diabetes mellitus	2.63	**0.043**	2.52	1.03–6.15
*H. pylori* positive	1.58	0.202	1.71	0.75–3.89
Peptic ulcer history	2.36	**0.044**	2.34	1.02–5.37
Upper abdominal pain	−0.19	0.342	0.67	0.30–1.52
Heartburn	−0.65	0.615	0.78	0.29–2.07
Nausea/vomiting	1.26	0.192	1.92	0.72–5.12
Bloating	1.79	0.074	2.10	0.93–4.74
Smoking^b^	0.86	0.237	2.40	0.56–10.23
Alcohol consumption^c^	2.58	**0.018**	3.00	1.20–7.48

^a^Crude *p* values obtained from Wald's test.

Response variable: presence of gastroduodenal ulcer in patients taking LDA.

OR: odds ratio.

CI: 95% confidence interval.

M: male, F: female.

^b^Over 5 cigarettes/day.

^c^More than 2 units/day, 1 unit: 10 mL pure alcohol.

NSAIDs: nonsteroidal anti-inflammatory drugs.

**Table 3:** The final multivariable logistic model.

Variables	*b* ^a^	SE	*p* ^b^	Adjusted OR	95% CI
Male gender	1.65	0.55	**0.003**	**5.20**	**1.77–15.34**
Anticoagulants	1.58	0.66	**0.017**	**4.85**	**1.33–17.68**
NSAIDs	2.08	0.68	**0.002**	**8.00**	**2.09–30.58**
Peptic ulcer history	1.12	0.54	**0.038**	**3.07**	**1.06–8.86**
Cerebrovascular disease	1.98	0.84	**0.018**	**7.27**	**1.40–37.74**
Liver disease	0.82	0.52	0.118	**2.27**	**0.81–6.35**
Constant	−3.69	0.72	**<0.001**	0.02	0.006–0.10

^a^Estimated unstandardized regression coefficients.

SE: standard error.

^b^Wald's test adjusted *p* value.

NSAIDs: nonsteroidal anti-inflammatory drugs.

**Table 4:** Assessment of model performance.

Performance indices	Final multivariable model
*Global measures of goodness-of-fit*	
Brier coefficient	0,15
*R* ^2^ (Nagelkerke)	0,43
Hosmer-Lemeshow goodness-of-fit test	*χ* ^2^ = 5,26, df = 8, *p* = 0,73
*Comparison of nested models tested *	
Likelihood ratio test (full model versus constant model)	*χ* ^2^ = 50,98, df = 15, *p* < 0,001
Likelihood ratio test (full model versus reduced model)	Δ*χ* ^2^ = 10,56, Δdf = 9, *p* = 0,307
*Discrimination*	
*C* stat (95% CI: lower-upper limit)	0,85 (95% CI: 0,79–0,92)
Somers' *D* index	0,71
Discrimination slope	1,00

*Note.* Full model: model with all potential candidates from univariate regression analysis.

Constant model: null model.

Reduced model: final model.
